# Three-button coronary reimplantation during valve-sparing aortic root replacement for giant cell arteritis with left main coronary artery aneurysm

**DOI:** 10.1016/j.xjtc.2026.102480

**Published:** 2026-06-04

**Authors:** Reiko Kanno, So Izumi, Hatsue Ishibashi-Ueda, Takuro Tsukube, Yutaka Okita

**Affiliations:** a Division of Cardiovascular Surgery, Japanese Red Cross Kobe Hospital and Hyogo Emergency Medical Center, Kobe, Japan; b Department of Diagnostic Pathology, Hokusetu General Hospital, Takatsuki, Japan; c Division of Cardiovascular Surgery, Cardio-Aortic Center, Takatsuki General Hospital, Takatsuki, Japan

**Figure:**
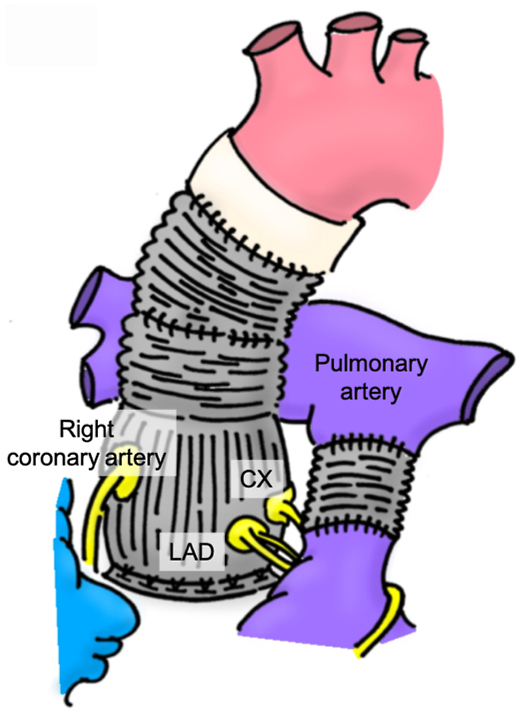
Three-button coronary reimplantation after left main aneurysm resection.

**Central Message**

Separate reimplantation of the LAD and circumflex arteries can enable an anatomic 3-button reconstruction when a left main aneurysm precludes a conventional left coronary button.

Giant cell arteritis (GCA) involves medium- and large-sized arteries and may affect the thoracic aorta, but annuloaortic ectasia (AAE) with a left main coronary artery (LMCA) aneurysm is exceptionally uncommon. We report valve-sparing aortic root replacement (VSRR) with complete LMCA aneurysm resection and 3-button coronary reimplantation, in which the left anterior descending (LAD) and circumflex arteries were reconstructed separately.

## Case Report

This work received institutional review board approval of Japanese Red Cross Kobe Hospital (date and number of approval: no. 26-473, May 15, 2026), and written informed consent was obtained from the patient for publication of this report and accompanying images.

**Figure 1:**
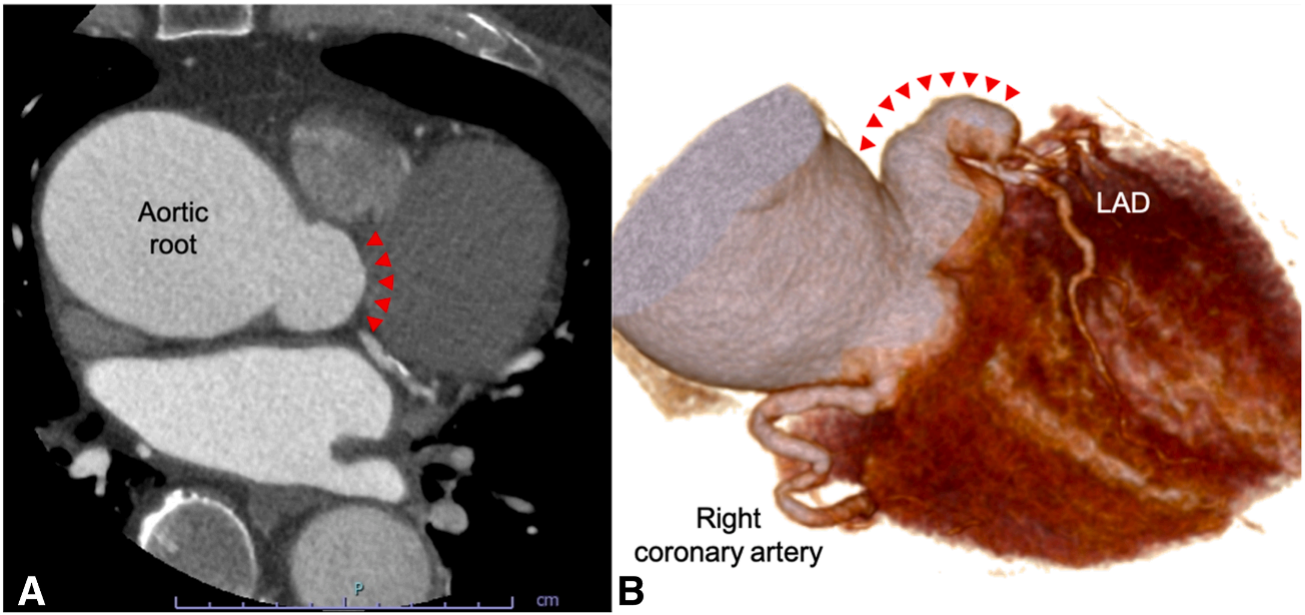
Preoperative computed tomography showing annuloaortic ectasia (A) and a left main coronary artery aneurysm extending to the bifurcation (B). *LAD*, Left anterior descending artery. The *arrowhead* symbol represent the LMCA aneurysm.

A 55-year-old man presented with heart failure caused by AAE and aortic regurgitation. Contrast-enhanced computed tomography showed a 60-mm sinus of Valsalva, a 65-mm ascending aorta, and a 15-mm LMCA aneurysm extending to the bifurcation (Figure 1). Echocardiography demonstrated a tricuspid aortic valve and moderate-to-severe aortic regurgitation attributable to incomplete leaflet coaptation.

**Figure 2:**
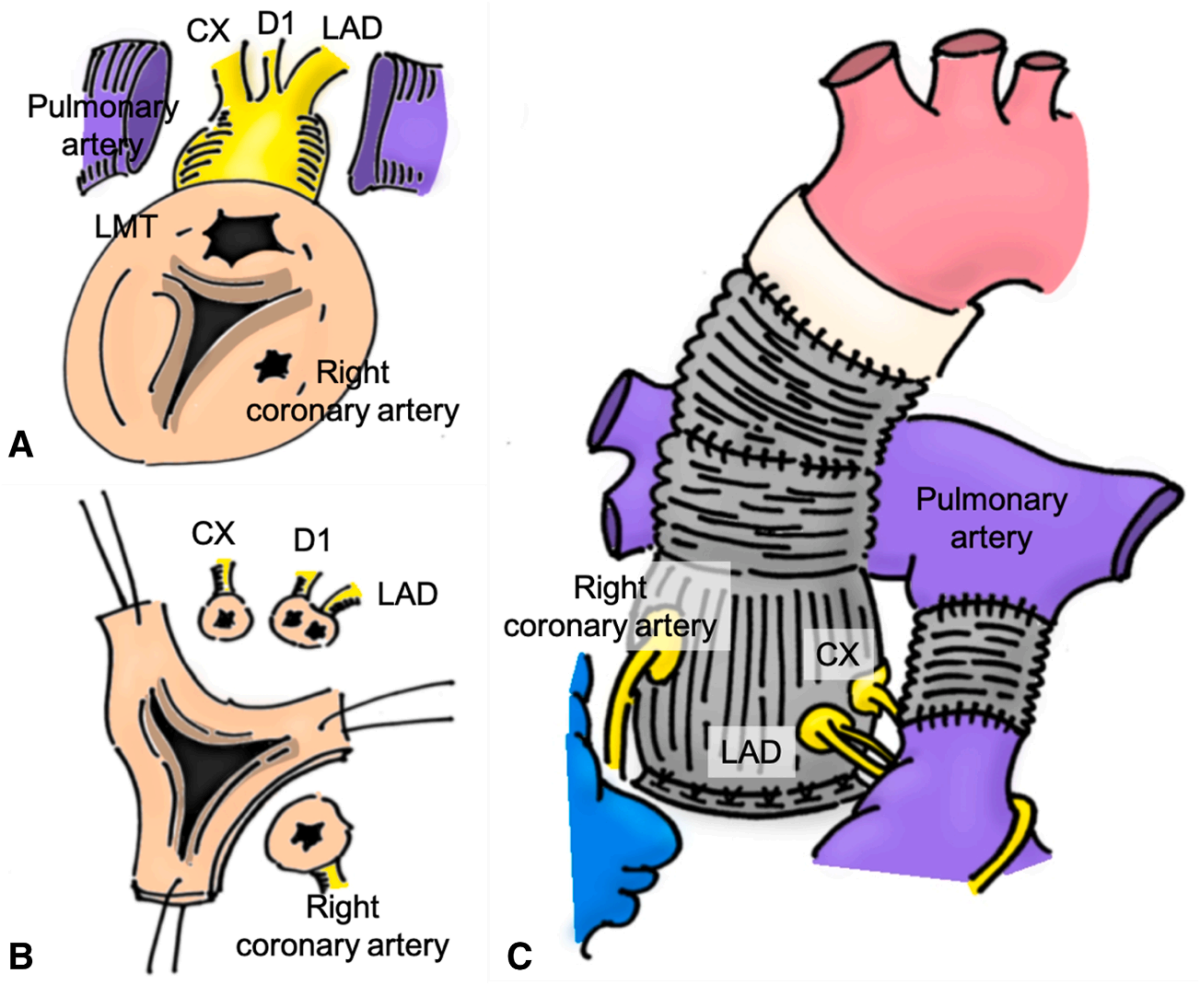
Operative schema. A, Pulmonary artery transection exposed the left main coronary artery aneurysm. B, The LAD and circumflex orifices were fashioned as separate buttons. C, Valve-sparing root replacement was completed with right coronary, LAD, and circumflex button reimplantation. *LAD*, Left anterior descending artery; *CX*, circumflex artery; *D1*, first diagonal branch; *LMT*, left main trunk.

VSRR was performed using a 28-mm Gelweave Valsalva graft (Terumo Aortic) with concomitant ascending aortic replacement. Intraoperatively, the aortic root was adherent to surrounding tissue, and the adventitia appeared pale and fibrotic, suggesting aortitis. After transection of the main pulmonary artery, the LMCA aneurysm and distal branches were adequately exposed (Figure 2, *A*). The aneurysm was completely resected. Because the aneurysm involved the LMCA bifurcation, the LAD and circumflex orifices were separated and fashioned as independent coronary buttons; the first diagonal branch was included in the LAD button because of its close proximity (Figure 2, *B*). After maximal mobilization of the proximal LAD and circumflex arteries, each button was directly reimplanted into the left sinus portion of the Valsalva graft using 5-0 polypropylene suture, together with the right coronary button (Figure 2, *C*; Video 1). The distal ascending aortic anastomosis was completed under deep hypothermic circulatory arrest with retrograde cerebral perfusion. The transected main pulmonary artery was reconstructed with a short 26-mm Gelweave graft to avoid coronary compression. The total operation time was 10 hours, with extracorporeal circulation time of 340 minutes, aortic crossclamp time of 279 minutes, and circulatory arrest time of 16 minutes.

**Figure E1:**
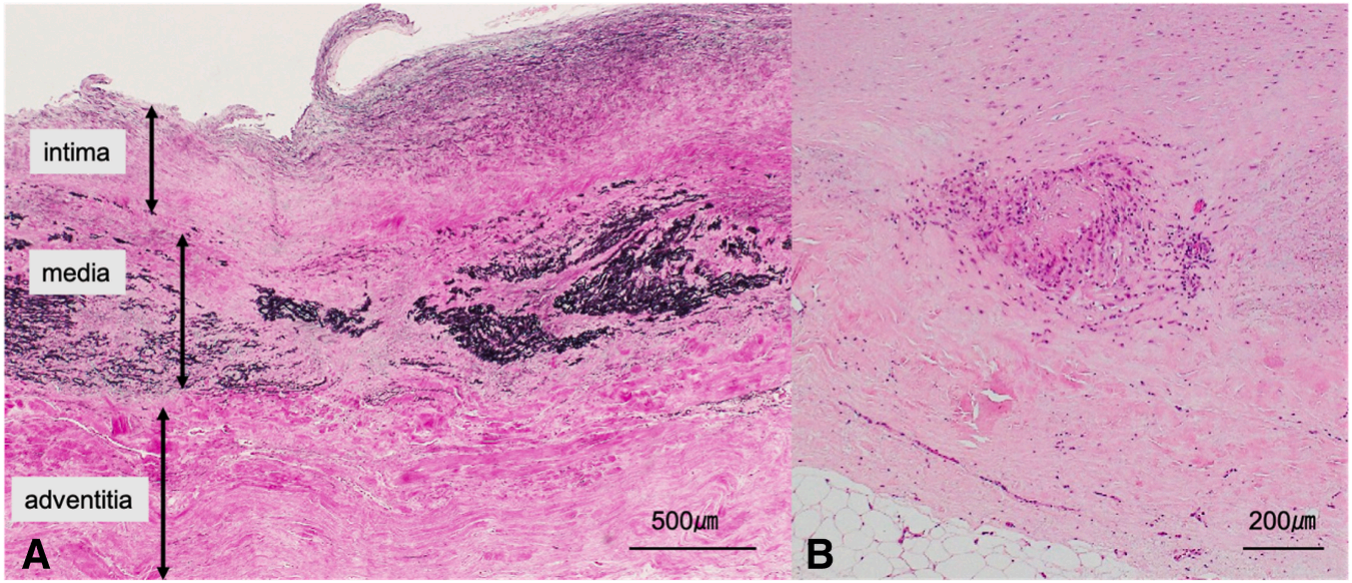
Histopathology of the aortic root. A, Disruption and fragmentation of the medial elastic lamina with marked thickening of the intima and adventitia (Elastica-van-Gieson stain; scale bar: 500 μm). B, Granulomatous inflammation with lymphocytes and multinucleated giant cells in the medial layer (hematoxylin and eosin stain; scale bar: 200 μm).

**Figure E2:**
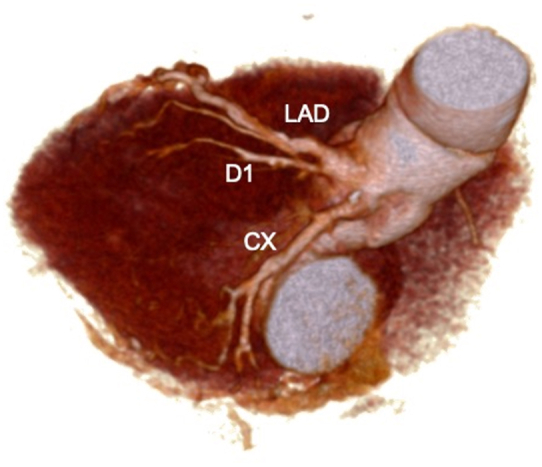
Postoperative computed tomography showing the reconstructed aortic root and left coronary arteries. *LAD*, Left anterior descending artery; *D1*, first diagonal branch; *CX*, circumflex artery.

Pathologic examination revealed destruction of the medial elastic lamina, medial necrosis, and chronic granulomatous inflammation with multinucleated giant cells, confirming chronic extracranial GCA (Figure E1). Postoperative computed tomography showed a well-reconstructed aortic root and patent left coronary reconstruction (Figure E2). Echocardiography demonstrated minimal aortic regurgitation and normal cusp motion. Corticosteroid therapy was initiated postoperatively, and the patient was discharged without complications on postoperative day 24. At 12 months, he remained asymptomatic on corticosteroid therapy; computed tomography and echocardiography showed patent coronary and pulmonary artery reconstructions, no LMCA aneurysm recurrence, and no recurrent aortic regurgitation.

## Comment

Coronary involvement in GCA is uncommon and usually stenotic; aneurysmal LMCA involvement is particularly rare.^1^ Standard treatment of LMCA aneurysms includes resection or exclusion with coronary artery bypass grafting.^2^ During aortic root replacement, interposition graft reconstruction, including Dacron graft reconstruction of the left coronary system, may be a simpler alternative.^3^ In this patient, however, direct 3-button reconstruction was selected because the LAD and circumflex origins were suitable for separate button creation and tension-free reimplantation, enabling complete aneurysm resection while preserving an anatomic coronary reconstruction.

Key technical points were pulmonary artery transection for exposure, complete LMCA aneurysm resection, and separate LAD and circumflex button reimplantation. The pulmonary artery was reconstructed with a short prosthetic graft because it was not diseased and was transected only for exposure. Potential pitfalls include coronary injury, bleeding, coronary distortion or compression, and prolonged myocardial ischemic time. Thus, this technique should be reserved for highly selected patients in whom complete aneurysm resection and tension-free anatomic reconstruction are feasible.

The durability of VSRR in GCA-associated AAE remains uncertain. Nevertheless, VSRR may be reasonable in selected younger patients with preserved cusps if postoperative anti-inflammatory therapy and close imaging surveillance are ensured.^4^ Preoperative suspicion of vasculitis would not have changed the need for root reconstruction but would have emphasized careful tissue assessment and early anti-inflammatory management.

## Conclusions

Three-button coronary reimplantation with separate LAD and circumflex reconstruction may be a useful option when an LMCA aneurysm precludes conventional left coronary button reimplantation during VSRR.

## Conflict of Interest Statement

The authors reported no conflicts of interest.

The *Journal* policy requires editors and reviewers to disclose conflicts of interest and to decline handling or reviewing manuscripts for which they may have a conflict of interest. The editors and reviewers of this article have no conflicts of interest.

## Supplementary Data

Video 1 Three-button coronary reimplantation during VSRR, including preoperative computed tomography, pulmonary artery transection, LMCA aneurysm resection, separate LAD and circumflex button creation, direct reimplantation, and postoperative computed tomography. Video available at: https://www.jtcvs.org/article/S2666-2507(26)00287-7/fulltext.
